# Development of a low‐cost 3D‐printed phantom for end‐to‐end testing of model‐based dose calculation algorithms (MBDCAs) in high‐dose‐rate (HDR) brachytherapy

**DOI:** 10.1002/acm2.70885

**Published:** 2026-10-30

**Authors:** Sean Grace, Lin Wang, Yijian Cao, Lyu Huang

**Affiliations:** ^1^ Department of Physics and Astronomy Hofstra University Hempstead New York USA; ^2^ Northwell New Hyde Park New York USA; ^3^ Donald and Barbara Zucker School of Medicine at Hofstra/Northwell Hempstead New York USA; ^4^ Department of Radiation Medicine Northwell Health Lake Success New York USA

**Keywords:** 3D Printing, End‐to‐end validation, Film dosimetry, HDR brachytherapy, Model‐based dose calculation algorithm

## Abstract

**Background:**

Model‐Based Dose Calculation Algorithms (MBDCAs), such as AcurosBV, have improved dosimetric accuracy in high‐dose‐rate (HDR) brachytherapy by accounting for medium heterogeneities and variations in scatter conditions. However, their safe and effective clinical implementation requires thorough commissioning, of which end‐to‐end (E2E) validation using physical phantoms is an important component. Commercial anthropomorphic phantoms can be costly and may offer limited flexibility for customized testing configurations.

**Purpose:**

This study aims to design, fabricate, and evaluate a novel low‐cost, modular, 3D‐printed phantom for E2E validation of MBDCAs in HDR brachytherapy. The phantom was developed to provide a practical, measurement‐based tool for commissioning and quality assurance under controlled heterogeneous conditions, particularly in resource‐limited settings.

**Methods:**

A modular phantom incorporating heterogeneity plugs (moderate‐density, air cavity, and calcium carbonate) was 3D printed using Polylactic acid (PLA) on a Bambu X1 Carbon printer. EBT4 films were scanned on an Epson 10000XL flatbed scanner, calibrated and used for planar dose measurements. The phantom was scanned with a Siemens CT simulator. Treatment plans were generated using BrachyVision in Eclipse v16.1, with dose distributions calculated using both TG‐43 and AcurosBV algorithms. The plan was delivered with a GammaMed Plus iX afterloader. Film measurements were compared against calculated dose maps using gamma analysis and dose profile comparisons.

**Results:**

The 3D‐printed phantom demonstrated high dimensional fidelity (± 0.15 mm) and allowed for reproducible catheter placement and film positioning. AcurosBV calculations showed close agreement with film measurements, with gamma passing rates of 95% at 2%/2 mm criterion and 94.2% at 3%/1 mm criterion. Mean dose differences between AcurosBV and film were 1.7% ± 1.9% for the vertical profile and −0.5% ± 1.7% for the horizontal profile. Under the controlled heterogeneous configuration of the 3D‐printed phantom, TG‐43 predicted higher doses than film by 3.6% ± 1.2% and 8.2% ± 1.5% for the vertical and horizontal profiles, respectively. These differences reflect the combined influence of the non‐water‐equivalent phantom body and the incorporated heterogeneous inserts.

**Conclusions:**

This low‐cost, modular, 3D‐printed phantom provides a practical platform for E2E validation of MBDCAs under controlled heterogeneous conditions. Its customizable design may complement existing commissioning methods, particularly in resource‐limited settings.

## 1 INTRODUCTION

HDR brachytherapy represents a cornerstone in modern cancer treatment, offering a highly targeted approach to radiotherapy that enables the delivery of precise, escalated doses directly to tumor tissues while sparing adjacent healthy structures.^1^, ^2^ Unlike external beam radiotherapy (EBRT), HDR brachytherapy positions radioactive sources in close proximity to or within the tumor, leveraging rapid dose fall‐off to achieve an optimal therapeutic ratio. This approach is especially advantageous in treating malignancies situated near critical organs‐at‐risk (OARs), such as gynecological,^3^ prostate,^1^ breast,^4^ and head and neck (H&N) cancers,^5^ either as a standalone treatment or in combination with EBRT for dose escalation.^2^

Accurate dose calculations are critical in HDR brachytherapy for ensuring effective treatment while minimizing risks to surrounding tissues. Most treatment planning systems rely on the American Association of Physicists in Medicine (AAPM) Task Group 43 (TG‐43) formalism, a dosimetry protocol that simplifies dose calculations by assuming single‐source dose superposition in an infinite water medium.^6^ While TG‐43 has provided a practical foundation for dose calculations, it does not account for patient‐specific anatomical heterogeneities, such as bone, adipose tissues, or air cavities, nor does it consider non‐water equivalent applicators, including surface applicators or shielded cylinder applicators.^3^ These limitations can introduce clinically significant inaccuracies in calculated dose distributions, potentially resulting in dose differences of up to 10% in Tandem and Ovoid (T&O) treatments.^7^ Either underestimation or overestimation of dose to the target volume or adjacent organs at risk could compromise dose calculation accuracy and the reliability of treatment planning.

To address these limitations, MBDCAs have been introduced into clinical brachytherapy practice. Unlike the TG‐43 formalism, MBDCAs account for medium heterogeneities and variations in scatter conditions.^8^ One such implementation is the AcurosBV, a Grid‐based Boltzmann Solver (GBBS) developed by Varian Medical Systems (Palo Alto, CA) and released in 2009. AcurosBV uses energy, spatial, and angular discretization to calculate photon fluence and convert it to dose, incorporating macroscopic cross‐section data for photon interactions. For Iridium‐192 (Ir‐192) sources, it models effective point sources based on energy and angular photon intensity. AcurosBV provides accurate dose calculations that are comparable to Monte Carlo simulations while requiring substantially less computation time, enabling practical evaluation of tissue heterogeneities, applicator materials, and altered scatter conditions within routine clinical workflows.^9^, ^10^, ^11^ In addition, Siebert et al reported a target dose difference of 3% between TG‐43 and AcurosBV in H&N treatment.^5^ In HDR skin brachytherapy, where tissue‐air interfaces are more pronounced, differences as high as 15% have been observed.^6^, ^12^ Similarly, Hofbauer et al reported a dose difference of 8% for D10cc to the skin between TG‐43 and AcurosBV for breast treatment.^13^ These findings underscore the clinical importance of using MBDCAs to improve dosimetric accuracy in cases involving tissue heterogeneities, shielding materials, and limited scatter conditions.

Despite these advantages, safe clinical implementation of MBDCAs requires thorough and comprehensive commissioning.^14^, ^15^ Although AAPM Task Group 186 and 372 provide standardized test cases for commissioning MBDCAs, these cases do not provide a physical, measurement‐based assessment of the complete clinical workflow. Experimental validation using physical phantoms is therefore an important complementary component of MBDCA commissioning. Anthropomorphic phantoms are commonly employed for this purpose, as they replicate patient anatomy by incorporating materials of varying densities, such as bone, adipose tissue, and air, to mimic heterogeneities encountered in clinical scenarios.^16^ Dosimetric validation is typically performed using radiochromic films, thermoluminescent dosimeters (TLDs), or other detectors placed at known locations.^17^, ^18^, ^19^ These measurements allow for direct comparison between calculated and measured dose distribution, providing a benchmark for assessing algorithm performance. However, commercially available anthropomorphic phantoms are often expensive and may provide limited flexibility for customized testing configurations.

These financial and logistical limitations have motivated interest in more adaptable and cost‐effective phantom development methods. Three dimensional printing has emerged as a promising technology for the rapid, low‐cost production of customized phantoms.^18^, ^20^, ^21^ It offers precise control over geometry and internal structure, allowing for the fabrication of anatomically accurate models with tunable densities and various materials. By adjusting infill percentage, wall thickness, and material type, such as acrylonitrile butadiene styrene (ABS) or PLA, 3D‐printed phantoms can be tailored to provide various material types and anatomical features with high fidelity.^17^ This flexibility has the potential of developing complex, heterogeneous models that better fits clinical validation needs than their commercially available counterparts. Additionally, the low material and production costs make 3D printing an attractive option for resource‐limited settings.^13^, ^18^, ^20^

To address this gap, this study aimed to design and validate a novel, low‐cost, modular, 3D‐printed heterogeneous phantom for E2E testing of AcurosBV in HDR brachytherapy. The phantom incorporates controlled heterogeneities and uses EBT4 radiochromic film to enable direct comparison between calculated and measured dose distributions. By integrating imaging, treatment planning, dose calculation, delivery, and experimental verification, the proposed phantom complements the standardized test cases and provides an accessible platform for evaluating MBDCA performance, particularly in resource‐limited settings.

## 2 METHODS

### 2.1 Film calibration and scanning

To establish a reliable dose‐response relationship, twelve 3 × 3 cm EBT4 radiochromic films (Ashland Inc., Bridgewater, NJ) were calibrated prior to experimental irradiation. Each film was exposed to a 6 MV photon beam from a TrueBeam linear accelerator (Varian Medical Systems, Palo Alto, CA), covering a dose range from 0 to 12 Gy. The irradiation was performed at a depth of 5 cm within a solid water phantom with a source‐to‐surface distance (SSD) of 95 cm.

Following exposure, all films were allowed to develop for 24 h for polymerization. The films were scanned with the same orientation in the central region of the scanner bed using an Epson 10000XL flatbed scanner (Seiko Epson Corporation, Tokyo, Japan) under consistent scanning conditions (300 dpi, 48 bits, and no color correction). The red channel was selected for analysis. The scanned images were processed using RIT software, version 6.8.64 (Radiological Imaging Technology, Inc., Colorado Springs, CO, USA), with the pixel values plotted against the corresponding doses. A piecewise polynomial function was utilized in RIT to generate the dose‐response calibration curve, which was subsequently used for dose conversion in the experimental measurements.

### 2.2 Phantom design and fabrication

The 3D‐printed phantom was designed to support film measurements while accommodating essential components, including the 15G 32 cm Flexi‐Needles (Best Medical International, Inc, Springfield, PA). The design began with conceptual sketches to ensure an adequately large surface area for data acquisition, followed by a detailed three‐dimensional model developed in TinkerCAD (Autodesk, San Francisco, CA), a user‐friendly 3D design website that allows for structural refinement.

The printer's specifications, such as resolution, material compatibility, and dimensional accuracy, were evaluated to confirm the feasibility of the design. The printing parameters are listed in Table 1. Bambu X1 Carbon offers dual auto bed leveling, a build volume of 25.6 × 25.6 × 25.6 cm, wide range of filament materials, and superior printing accuracy. The finalized phantom, sized 5 × 8 × 8 cm^3^, was fabricated on the Bambu X1 Carbon with PLA filaments. The phantom was composed of three modular sections for easy assembly and potential replacement if reprinting was required. The structural features of each section are described below.:

- Bottom Section: The base section measured 8 cm (width) × 8 cm (length) × 1.2 cm (height) and contained six 2.1 mm diameter holes, each extending 7.9 cm in length, designed to guide the catheters through the phantom with precision.
- Middle Section: The middle block, measuring 8 cm × 8 cm × 1.6 cm, featured four 10 × 10 mm^2^ square cavities drilled to a depth of 7.9 cm. These cavities were designed to accommodate interchangeable inserts representing different heterogeneous conditions.:
  - Two PLA plugs were fabricated: one with 100% infill to provide a relatively high‐density (1.24 g/cc) printed material and another with 25% infill to provide a reproducible low‐density, structured heterogeneity.
  - A third cavity was left empty to create an air gap representative of air cavities that may be encountered clinically.
  - The fourth cavity housed a 10 × 10 mm^2^ case filled with calcium carbonate paste (prepared by mixing calcium carbonate with water), tapped gently during preparation to reduce trapped air and improve the uniformity of the material distribution.

**TABLE 1:** Printing parameters used for each phantom component in Bambu Studio.

Parameters	Settings
Infill Density (%)	100%/25%
Infill Pattern	Rectilinear/Grid
Nozzle size	0.4 mm
Nozzle Temp.	190°C
Speed	270 mm/s
Filament Material	PLA Basic
Filament Diameter	1.75 mm
Printing Plate	Textured PEI Plate
Plate Temp.	55°C

The air and calcium carbonate inserts were intended to represent more clinically relevant radiological heterogeneities, including air gaps and bone‐like structures.

- Top Section: The uppermost section was redesigned as a 0.5 cm thick plate with dimensions of 7.9 cm × 7.4 cm. This thinner design ensures a flat connection that sandwiches the EBT4 film securely, minimizing the risk of film bowing. To enhance pressure on the film and ensure consistent dose measurement, a backup catheter piece was added as additional weight. This backup piece measured 8 cm × 8 cm × 1.2 cm and featured six catheter holes, each with a 0.2 cm diameter. This also allows additional needle configurations and supports the creation of more complex treatment plans.

### 2.3 Phantom imaging and treatment planning

After assembly, the phantom was positioned on a Styrofoam block and imaged with a SOMATOM Definition AS CT simulator (Siemens Healthineers, Germany) to acquire high‐resolution anatomical data necessary for treatment planning. A slice thickness of 1 mm was used to capture detailed images, which were then imported into the Eclipse v16.1 treatment planning system.

The catheters were inserted into the pre‐defined holes in the phantom's bottom section, while the EBT4 film was placed in the slit at the top. A dummy source was used to facilitate accurate dwell positioning in the treatment planning system. Two CT scans were acquired: one with the dummy source in place (Figure 1) and one without. Image fusion was performed within Eclipse to accurately register the scans and establish clear spatial relationships.

**FIGURE 1:**
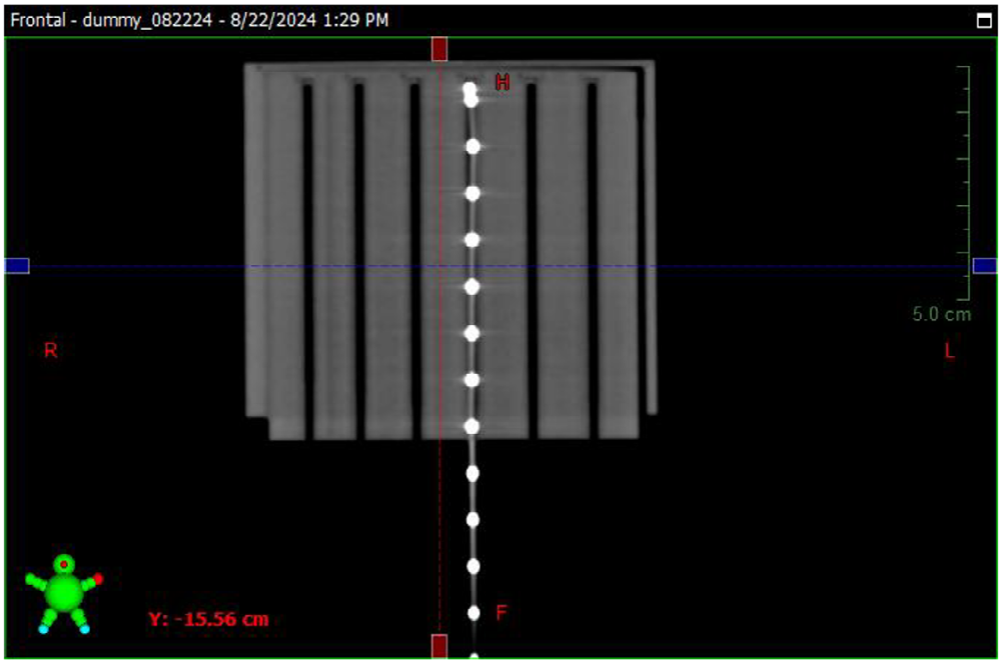
Visualization of the dummy source positioned within the 3D‐printed inhomogeneous phantom using the image registration workspace in Eclipse v16.1.

In Eclipse, the inhomogeneity plugs were contoured, and mean Hounsfield Unit (HU) values were calculated for each plug (Figure 2). A planning target volume (PTV) was delineated around the frame securing the EBT4 film. The prescribed dose was set to 600 cGy. Initial optimization was conducted using the TG‐43 dose calculation formalism, which assumes a homogenous medium for dose distribution. The plan was then recalculated with the AcurosBV (Figure 3). The algorithm assigns material properties on a voxel‐by‐voxel basis using the CT HUs, the institution‐specific CT to physical density calibration curve, and the internal material table. The finalized treatment plan was exported to the GammaMed Plus iX afterloader (Varian Medical Systems, Palo Alto, CA) for delivery.

**FIGURE 2:**
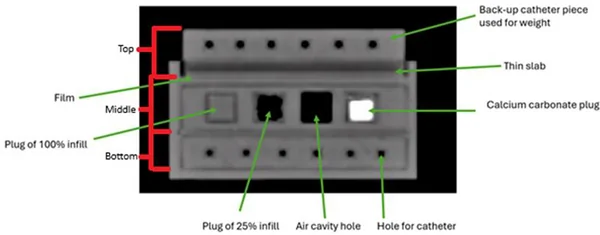
3D‐printed inhomogeneous phantom designed for E2E validation of AcurosBV using film dosimetry. The bottom section consists of the base slab containing the catheter channel pathways. The middle section contains interchangeable inhomogeneity inserts positioned at the measurement plane, including a 100% infill plug, 25% infill plug, air cavity, and calcium carbonate plug. Radiochromic film is placed at the top section for dose measurement. A thin compression slab secures the film and inserts in place, while a back‐up catheter segment positioned above provides consistent weighting and ensures reproducible film–insert contact during irradiation.

**FIGURE 3:**
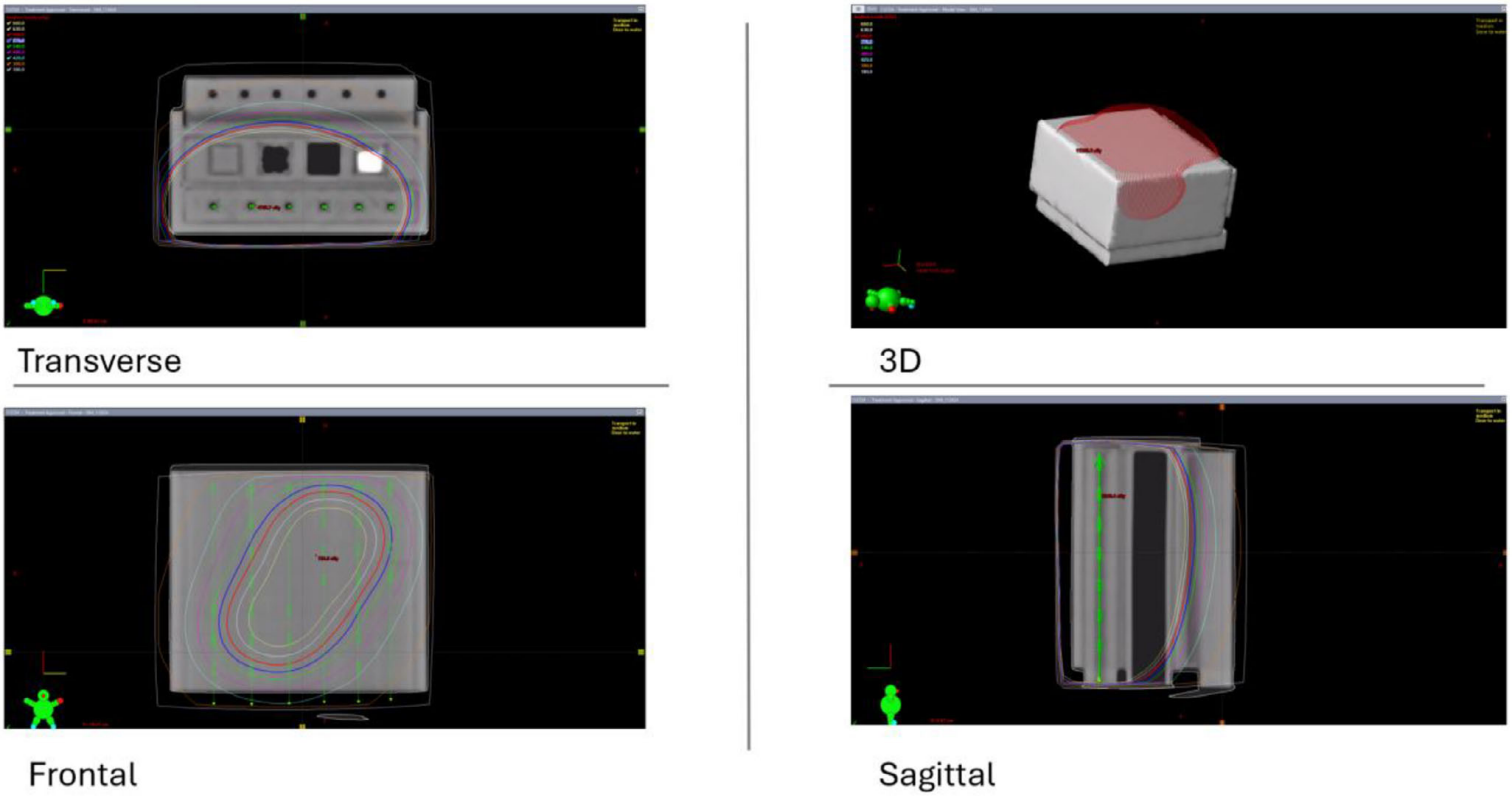
Visualization of the 3D‐printed inhomogeneous phantom in the brachytherapy treatment planning workspace of Eclipse v16.1. Representative transverse, frontal, sagittal, and 3D views show the phantom geometry, catheter pathways, inhomogeneity inserts, and planned dose distribution calculated using AcurosBV.

### 2.4 Irradiation setup

A 7.5 cm × 8 cm piece of EBT4 film was affixed atop the phantom. Six transfer tubes were utilized for connecting the catheters with the GammaMed plus IX afterloader. The fully assembled phantom, with catheters and EBT4 film in place, was irradiated according to the optimized treatment plan (Figure 4).

**FIGURE 4:**
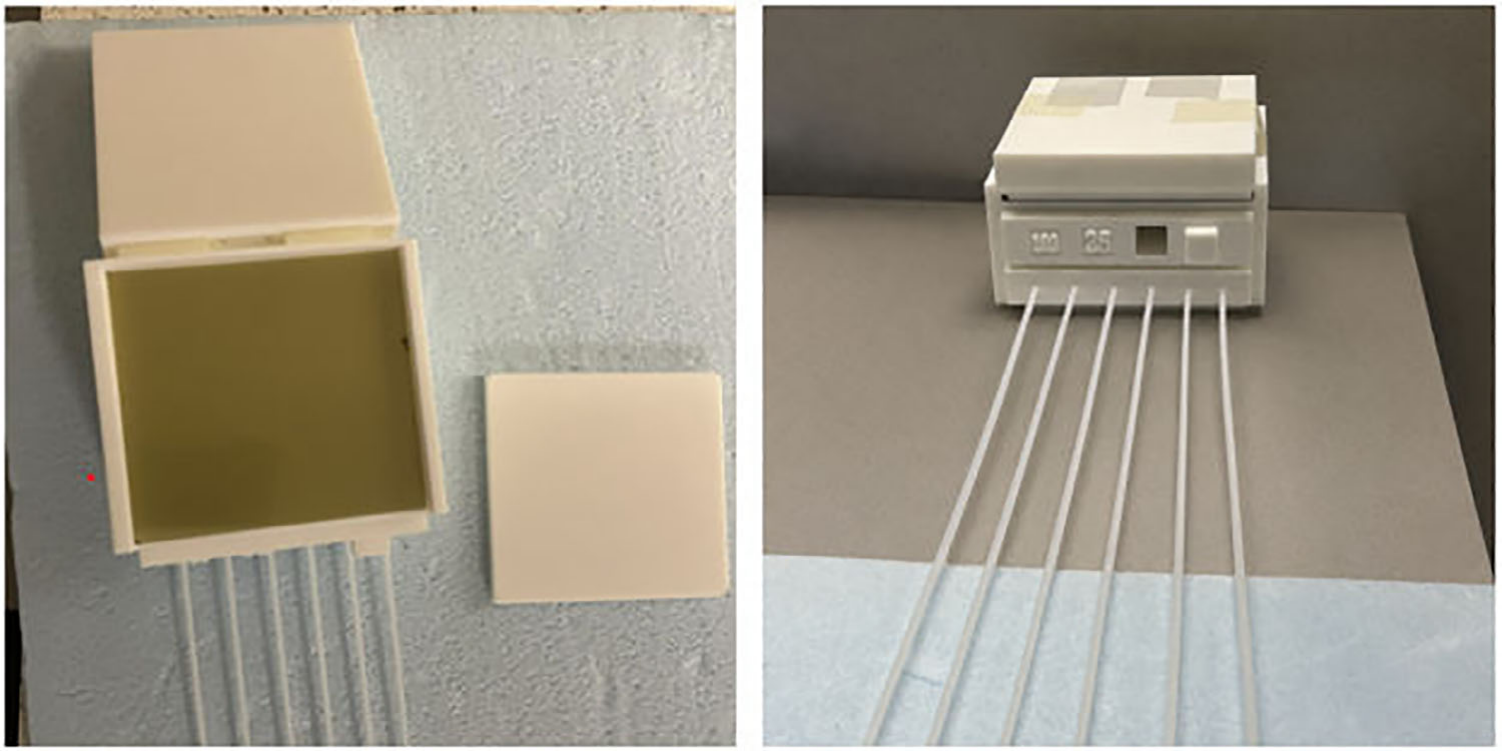
Experimental setup for E2E validation of AcurosBV using the 3D‐printed inhomogeneous phantom and film dosimetry. The interchangeable plugs are labeled according to infill level, with the printed number corresponding to one‐tenth of the infill percentage; for example, 100 indicates 100% infill and 25 indicates 25% infill. The unlabeled plug corresponds to the insert filled with calcium carbonate paste.

### 2.5 Dosimetric analysis

Following irradiation, the EBT4 film was scanned 24 h post‐exposure with the Epson 10000XL flatbed scanner using the same protocol in film calibration. The scanned images were analyzed using RIT version 6.8.64 software. The software used the red channel for film analysis and applies the calibration curve to convert the film`s pixel values into doses.

The gamma analysis is performed by first importing both the dose plane from the treatment planning system and the scanned film image into RIT.^22^ The two dose planes were registered with RIT's auto‐registration feature, which identifies common high‐dose or high‐gradient points between the two images and aligns them by applying shifts, rotations, and scaling transformations. The Eclipse exported dose plane was designated as the reference and the film measurement as the target. A region of interest (ROI) was selected in the relevant area for dose comparison. The two dose planes were both normalized to the max point dose of the reference dose plan. Gamma index was then computed for each pixel based on the dose difference and distance‐to‐agreement (DTA) setting between the reference and target images. If the point of interest doesn't exceed the pre‐set dose difference limits and falls within the DTA threshold, a gamma value of less than 1 is recorded, indicating an acceptable match.

The dose profile comparison was conducted between the AcurosBV‐calculated dose, the TG‐43‐calculated dose, and the film‐measured dose. Profiles were extracted from registered images in RIT to ensure alignment, enabling consistent export from the same plane. The profiles were taken along the central axis of the image for analysis. In Eclipse, the treatment plan was duplicated and recalculated using the TG‐43 formalism. The distance to the center of the film location was measured using Eclipse's measurement tool, and the dose profile tool was employed to extract dose coordinates along this axis. The collected data, including positional distances (in centimeters) and corresponding dose values, were plotted using a Python (Python Software Foundation, Beaverton, Oregon) script to facilitate a direct visual and quantitative comparison between the three dose distributions. Mean percent differences were calculated from point‐wise comparisons using

$$\frac{D_{\mathrm{comp}}-D_{\mathrm{ref}}}{D_{\mathrm{ref}}}\times 100\%$$

where the three comparisons were TG‐43 verses film, AcurosBV verses film, and TG‐43 versus AcurosBV. Thus, film served as the reference for the TG‐43 verses film and AcurosBV verses film comparisons, and AcurosBV served as the reference for the TG‐43 verses AcurosBV comparison. Reported uncertainties represent the standard error of the mean of the comparisons.

### 2.6 Uncertainty analysis

Measurement uncertainty was evaluated following the recommendations of the International Atomic Energy Agency (IAEA) TECDOC‐1585 and AAPM TG‐235,^23^, ^24^ which recommend identification of all significant uncertainty sources associated with radiochromic film dosimetry and treatment delivery with the 3D‐printed phantom. Individual uncertainty components were classified as Type A (evaluated by statistical analysis of repeated measurements) or Type B (evaluated from calibration certificates, manufacturer specifications, published literature, or engineering tolerances). The combined standard uncertainty was obtained by the root‐sum‐square (RSS) method assuming independent uncertainty components.

The methodology for estimating uncertainties associated with radiochromic film dosimetry followed that described by Sorriaux et al. and Huang et al.,^25^, ^26^ including contributions from scanner reproducibility, scanner spatial non‐uniformity, film non‐uniformity, and calibration curve fitting. These uncertainties have been extensively characterized for radiochromic film dosimetry and therefore were adopted from previously validated methodologies rather than remeasured in this study.

Additional uncertainty sources specific to the present E2E workflow included phantom fabrication, component assembly, film positioning, CT image resolution, needle and source positioning, treatment delivery, and registration between the planned dose plane and the measured film doses. The dimensional accuracy of the fabricated phantom was assessed to be ± 0.15 mm using caliper measurements across repeated prints. The influence of geometric uncertainties on dose depends on the local dose gradient. Accordingly, uncertainties related to phantom fabrication, needle positioning, film placement, dwell position, and image registration were propagated to dose using the local spatial dose gradient. These contributions are expected to be most important near the source.

Film positioning uncertainty resulted from placement of the EBT4 film between modular phantom components and from small differences in assembly before irradiation. The phantom design minimized film displacement and bowing by maintaining a flat interface and applying pressure with the upper component. Dose calculation uncertainty associated with AcurosBV was included using previously published estimates of approximately 1% for MBDCAs.^12^, ^27^ Similarly, the uncertainty in Ir‐192 source strength calibration was taken from AAPM TG‐138 recommendations, while uncertainty associated with dwell position accuracy was obtained from published HDR brachytherapy commissioning studies.^28^, ^29^

Registration between calculated and measured dose distributions introduced an additional uncertainty in the gamma and profile analyses. Although automatic registration was used, residual translational or rotational misalignment may produce local dose differences, particularly in high‐gradient regions. CT voxel size and contouring resolution also contributed to the geometric uncertainty in material boundaries, and extraction of the calculated dose plane.

## 3 RESULTS

### 3.1 Calibration curve

The dose‐response calibration curve for the EBT4 film is presented in Figure 5. The x‐axis represents the dose in cGy, while the y‐axis indicates the pixel values obtained from the red channel of the scanned film images. The curve indicates a consistent decrease in pixel values with increasing dose. This trend is attributed to increased polymerization at higher radiation, which results in greater light absorption. This calibration curve was subsequently used to convert pixel values to dose values in experimental measurements.

**FIGURE 5:**
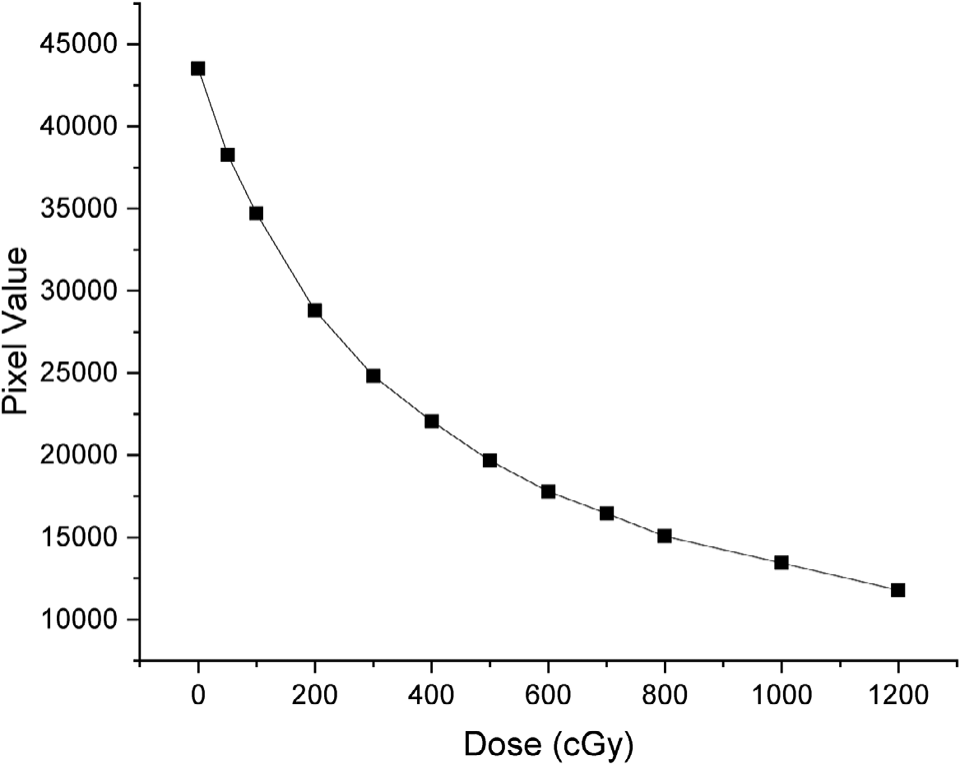
Film calibration curve derived from scanned radiochromic film measurements.

### 3.2 Printing accuracy

The Bambu X1 Carbon 3D printer demonstrated a dimensional accuracy of ± 0.15 mm, as verified by caliper measurements. Repeated fabrication of the phantom produced consistent dimensions, supporting the reproducibility of the manufacturing process and its suitability for generating detailed phantom geometries. This accuracy is achieved through several advanced features, including a 7 µm Lidar sensor that enhances layer alignment precision and a dual auto bed leveling system to maintain consistent print calibration. Additionally, active vibration compensation (XY) and extrusion compensation contribute to print smoothness and dimensional stability, even in complex structures. These technological advancements collectively facilitated the reliable fabrication of an intricate phantom structure, supporting stable and precise positioning of both film and catheters for dosimetric analysis.

### 3.3 Treatment planning and dose delivery

The treatment plan utilized six needles with specific dwell position configurations. The first and third channels each had three dwell positions, spaced 1 cm apart, starting from the end of the channel. The second and fifth channels had six dwell positions, spaced 1 cm apart, with the last dwell position stopping 7 cm into the channel. The fourth and sixth channels each had three dwell positions, starting 4 cm from the end of the channel, with 1 cm spacing between positions. The total planned dwell time was 869.8 s at a nominal 10 Curie source (Figure 4). The actual delivery time was extended to accommodate the actual source activity of our GammaMedplus iX afterloader.

The 3D‐printed phantom contained plugs with varying HUs and relative electron densities to simulate inhomogeneous materials:

- Dense Plug: Calcium carbonate filled: Average HU of 1305.8 ± 718.5 with a mean mass density of 1.91 g/cc, and a mean relative electron density of 1.77.
- High Density PLA Plug (100% fill): Average HU of 107.9 ± 55 with a mean mass density of 1.11 g/cc, and a mean relative electron density of 1.06.
- Low Density PLA Plug (25% fill): Average HU of −413.3 ± 373.4 with a mean mass density of 0.58 g/cc, and a mean relative electron density of 0.57.
- Low Density (Air Cavity): Average HU of −974.7 ± 109.6 with a mean mass density of 0.03 g/cc, and a mean relative electron density of 0.03.

These variations provided a controlled heterogeneity test for the dose calculation algorithms, with a vacant plug slot intentionally left as an air cavity to introduce additional heterogeneity.

### 3.4 Gamma analysis

Gamma analysis demonstrated close agreement between the AcurosBV calculations and film measurements (Figure 6). At a 2%/2 mm gamma criterion, the passing rate was 95%. Altering the criterion to 3%/1 mm yielded a passing rate to 94.2% (Table 2). This result supports the suitability of the proposed phantom for algorithm verification.

**FIGURE 6:**
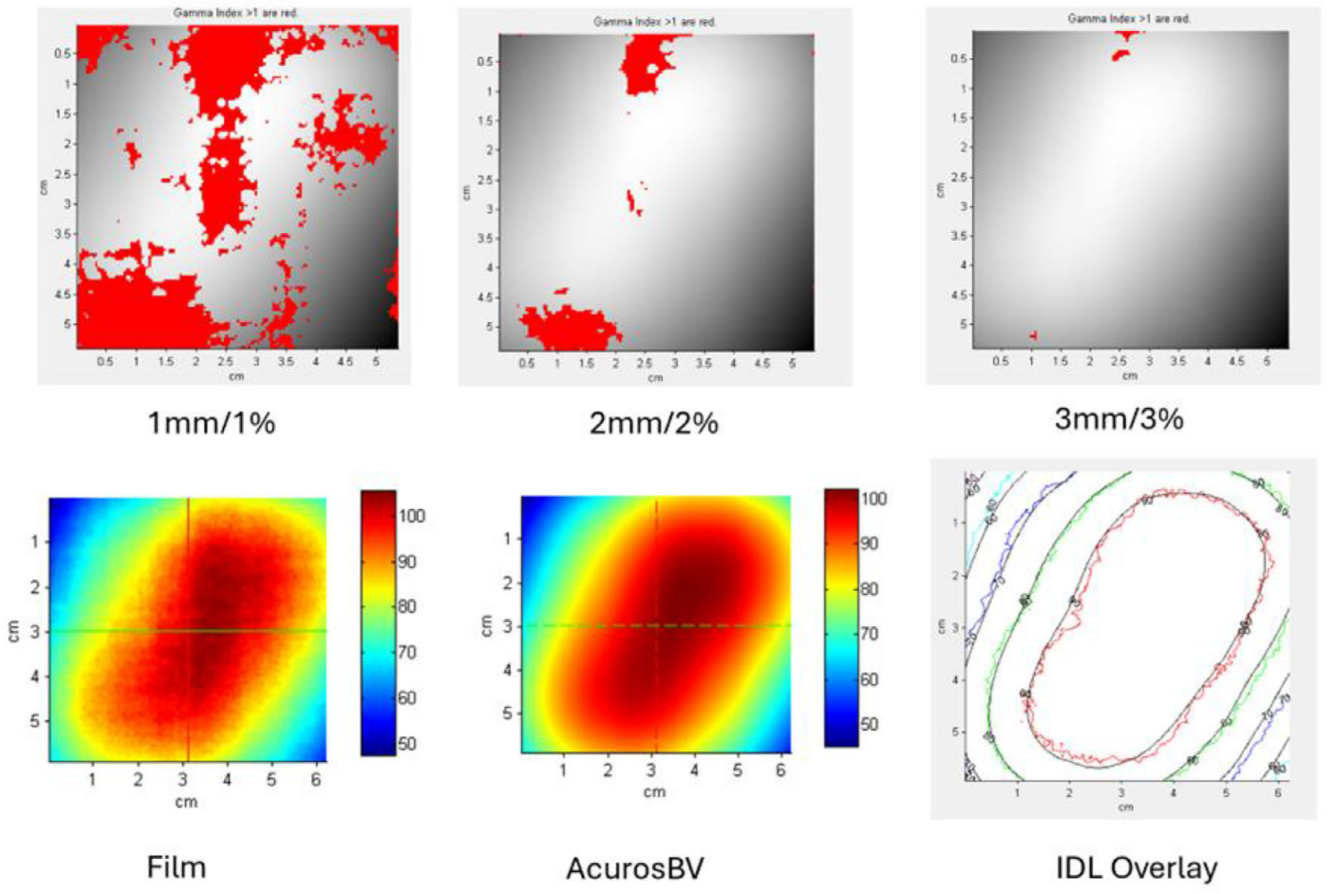
Two‐dimensional dose distribution comparison between EBT4 film measurements and AcurosBV dose calculations. The EBT4 film measurement is shown as the target dose plane, and the AcurosBV calculation is shown as the reference dose plane, with each presented using different display scales. The lower‐right panel displays the overlaid isodose lines for the reference and target dose distributions. The top row summarizes the gamma analysis results.

**TABLE 2:** Summary of gamma pass rates across the evaluated dose difference and distance‐to‐agreement gamma criteria between AcurosBV and film.

	Dose diff. (%)				
DTA (mm)	1	2	3	4	5
**1**	65.0%	82.6%	94.2%	98.2%	99.5%
**2**	87.9%	95.0%	98.6%	99.7%	100.0%
**3**	95.5%	98.7%	99.8%	100.0%	100.0%

### 3.5 Dose profile comparison

Dose profile comparisons provided further quantitative insight (Table 3). The comparison between AcurosBV and film measurement revealed a mean difference of 1.7% ± 1.9% in the vertical orientation (in plane with the heterogeneity plugs at the center of the phantom) as seen in Figure 7, and a mean difference of −0.5% ± 1.7% in the horizontal orientation (orthogonal to the heterogeneity plugs at the center of the phantom). In contrast, TG‐43‐calculated profiles showed larger deviations, which shows 8.2% ± 1.5% and 3.6% ± 1.2% in the horizontal and vertical orientation, respectively.

**TABLE 3:** Mean percent differences between dose profiles for EBT4 film measurements, TG‐43 calculations, and AcurosBV calculations along the vertical and horizontal orientations.

	TG‐43 vs Film	AcurosBV vs Film	TG‐43 vs AcurosBV
**Vertical**	3.6% ± 1.2%	1.7% ± 1.9%	5.1% ± 1.3%
**Horizontal**	8.2% ± 1.5%	− 0.5% ± 1.7%	10.2% ± 0.5%

Mean percent differences were computed from point‐wise comparisons as $\frac{D_{\mathrm{comp}}-D_{\mathrm{ref}}}{D_{\mathrm{ref}}}\times 100\%$, where the evaluated comparisons were TG‐43 versus film, AcurosBV versus film, and TG‐43 versus AcurosBV. For each comparison, the second method listed was used as the reference. Uncertainties are reported as the standard error of the mean.

**FIGURE 7:**
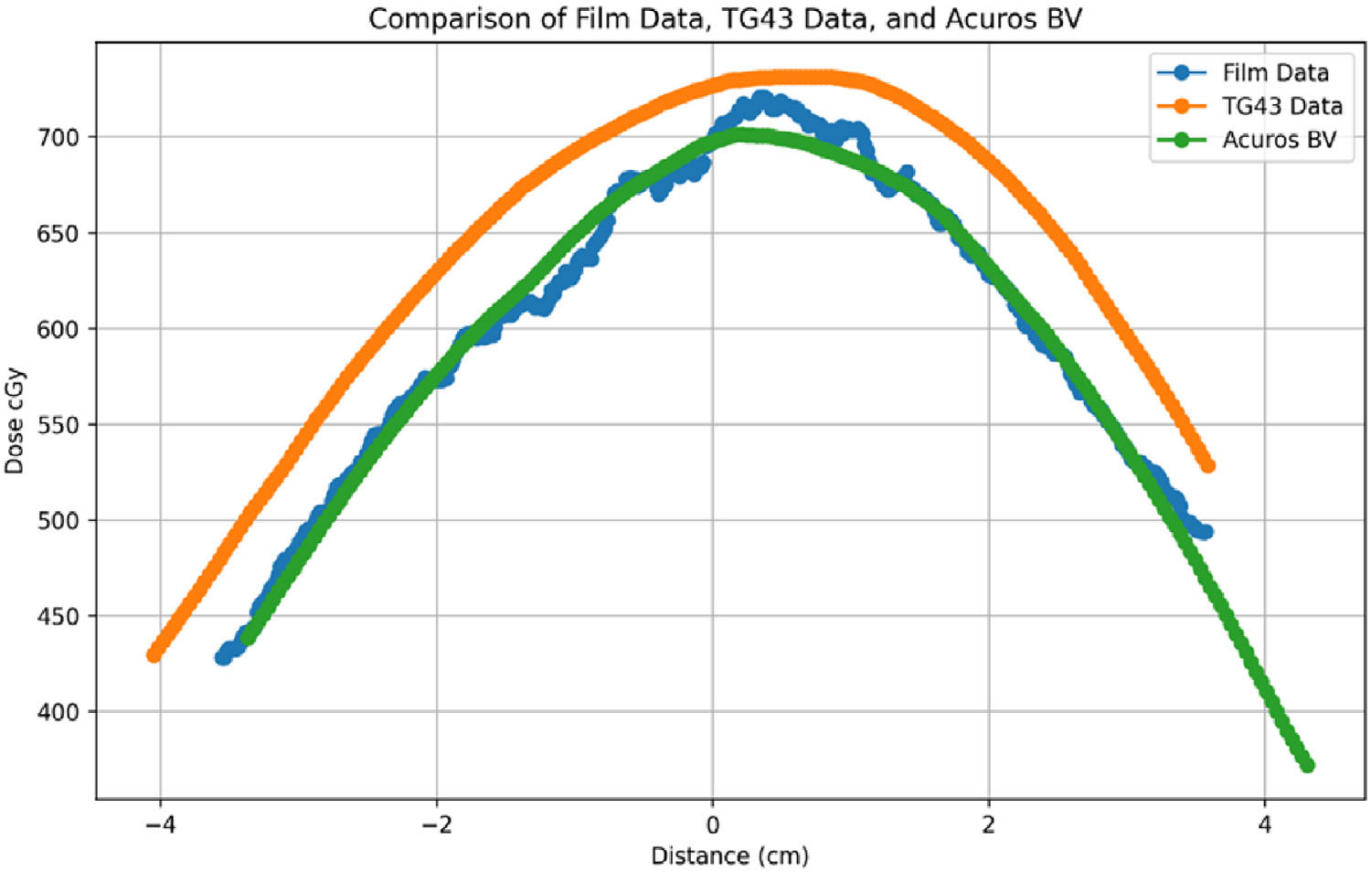
Comparison of dose profiles obtained from EBT4 film measurements, AcurosBV dose calculations, and TG‐43 dose calculations. This is a horizontal profile taken at the center of the film perpendicular to the direction of the heterogeneity plugs.

### 3.6 Uncertainty budget

The estimated uncertainty budget for the E2E validation with film dosimetry and 3D‐printed phantom is summarized in Table 4. Individual uncertainty components were classified as either Type A or Type B uncertainties following the recommendations of the Guide to the Expression of Uncertainty in Measurement (GUM), IAEA TECDOC‐1585, and AAPM TG‐235.^23^, ^24^, ^30^ The combined Type A uncertainty was 1.36% (k = 1), while the combined Type B uncertainty was 2.98% (k = 1). Combining these independent components by the root‐sum‐square method resulted in a combined standard uncertainty of 3.28% (k = 1) and an expanded uncertainty of 6.56% (k = 2), corresponding to an approximate 95% confidence level. The largest contributors to the overall uncertainty were the film calibration curve, Ir‐192 source strength calibration, AcurosBV dose calculation, dwell position accuracy, and geometric effects related to phantom fabrication.

**TABLE 4:** Uncertainty budget for end‐to‐end validation of AcurosBV calculations with the proposed 3D‐printed phantom and film dosimetry.

Source	Type	Estimated uncertainty (%)	Basis
Scanner reproducibility	A	0.2–0.3	Repeated scans under identical conditions
Scanner spatial nonuniformity	A	0.3–0.5	Measurements at multiple scanner‐bed positions
Intrasheet film nonuniformity	A	0.5	Measurements from different regions of the same film shee
Intersheet film nonuniformity	A	0.8	Measurements from different film sheets
Calibration curve fitting	B	1.5	Uncertainty associated with the RIT polynomial calibration fit
Film handling and orientation	B	0.5	Standardized film handling and scanning protocol
Linac‐based film calibration	B	0.9	Published uncertainty for linac‐based film calibration
Ir‐192 source strength	B	1.3	Source‐strength uncertainty based on AAPM TG‐138
AcurosBV dose calculation	B	1	Published uncertainty associated with the dose‐calculation algorithm
Dwell position accuracy	B	1	HDR afterloader commissioning and positional tolerance
CT voxel size and contouring	B	0.5	CT image resolution and structure delineation
Phantom dimensional accuracy	B	0.75–1.0	Caliper‐verified manufacturing tolerance of ± 0.15 mm
Phantom assembly / film positioning	A	0.5	Repeated phantom assembly and film placement
TPS‐to‐film registration	A	0.5–1.0	Repeatability of image registration

Individual uncertainty components were categorized as Type A or Type B uncertainties and combined using the root‐sum‐square method. The resulting combined standard uncertainty was 3.28% (k = 1), with an expanded uncertainty of 6.56% (k = 2).

The reported combined uncertainty should therefore be interpreted as an estimate of the overall uncertainty in the E2E validation rather than as a uniform point‐by‐point uncertainty across the entire dose plane. In particular, local uncertainty may exceed the reported average value in regions of steep dose gradient

## 4 DISCUSSION

The results of this study demonstrate the feasibility of using 3D printing to develop a low‐cost, modular phantom for E2E validation of MBDCAs in HDR brachytherapy. The proposed phantom was designed to evaluate the complete workflow, including CT imaging, treatment planning, dose calculation, delivery, and film‐based measurement, under controlled heterogeneous conditions. The phantom demonstrated dimensional accuracy within ± 0.15 mm, as verified by caliper measurements, and repeated fabrication produced consistent component dimensions. These characteristics are essential for ensuring consistent catheter and plug placement, as well as dosimeter positioning, which are the key parameters for consistent dosimetric measurements.

The modular design enabled the incorporation of inserts with different physical densities and material compositions. The air cavity and calcium‐carbonate insert were selected to represent radiological conditions that may be encountered clinically, including air gaps and bone‐like heterogeneities. In contrast, the low‐ and high‐density PLA inserts, with measured physical densities of approximately 0.50 and 1.11 g/cm^3^, respectively, were not intended to reproduce specific biological tissues. Rather, it was designed as a reproducible, structured heterogeneity for testing algorithm performance under conditions that differ substantially from water. Similarly, the PLA phantom body is not water equivalent and was used as a practical, non‐water‐equivalent medium for testing the ability of the algorithm to account for material composition and heterogeneity. The modular configuration nevertheless enables users to fabricate additional inserts with alternative densities, materials, geometries, or detector accommodations, including inserts designed for TLDs or OSLDs, according to local commissioning and quality assurance needs.

The gamma analysis and dose profile comparisons demonstrated close agreement between AcurosBV calculations and EBT4 film measurements in the proposed heterogeneous phantom. Gamma passing rates of 95% at 2%/2 mm and 94.2% at 3%/1 mm, along with an average profile dose difference below < 2%, support the ability of AcurosBV to calculate dose in the tested heterogeneous environment. Multiple gamma criteria were reported in this study to allow readers to interpret the findings in the context of their institutional practices. The observed agreement between AcurosBV and film is consistent with previous investigations conducted under heterogeneous conditions. Boman et al. reported close agreement between Acuros BV calculations and film measurements, with dose discrepancies within ∼1.5% at clinical depths in superficial mold treatments, while TG‐43 overestimated dose by up to ∼15% in the absence of full scatter conditions.^12^ Howie et al. similarly showed that TG‐43 overestimates dose across a large cohort of breast brachytherapy cases, particularly near low‐density regions such as skin and lung.^31^ Collectively, these findings support the importance of accounting for material composition, and altered scatter conditions when evaluating dose distributions in HDR brachytherapy.

In the present study, TG‐43 generally predicted higher doses than those measured with film. This difference must be interpreted in the context of the phantom composition. The main body of the phantom was fabricated from PLA, which is not water equivalent and therefore does not reproduce the unbounded homogeneous water medium assumed by TG‐43. PLA was selected because it is inexpensive, widely available, relatively safe to print, and consistent with the objective of developing an accessible phantom for resource‐limited settings. Its non‐water‐equivalent composition also created a deliberately sensitive test condition in which the heterogeneity correction capabilities of AcurosBV could be evaluated against physical measurements. Consequently, the differences observed between TG‐43 and AcurosBV reflect the combined effects of the PLA phantom body and the intentionally introduced heterogeneous plugs. These results should not be interpreted as a general comparison of the two calculation algorithms across all clinical conditions. Smaller differences would be expected if the phantom were fabricated from a water‐equivalent material. Although TG‐43 dose overestimation may have clinical implications in some treatment geometries,^31^, ^32^ the present study was not designed to quantify such effects in patients or to establish the clinical superiority of one algorithm over another. The primary objective was to determine whether the proposed phantom could support practical E2E validation of AcurosBV in a heterogeneous environment. Within this defined scope, the closer agreement between AcurosBV and film indicates that the algorithm accounted for the material properties and geometry of the fabricated phantom under the tested conditions. The phantom therefore could complement the standardized test cases described in TG‐186 and TG‐372. Those test cases support comparison of locally implemented MBDCAs with reference Monte Carlo and TG‐43 results under defined conditions. The proposed phantom adds a physical experimental assessment of the complete local workflow, including imaging, reconstruction, treatment planning, dose calculation, delivery, and dose verification.

The EBT4 radiochromic film was selected because of its high spatial resolution and suitability for measuring steep dose gradients.^12^ The red channel was chosen because it offers the highest sensitivity for dose measurements within the evaluated dose range and corresponds closely to the principal absorption region of the film's active layer.^23^ Film calibration was performed using a 6 MV photon beam in accordance with the recommendations of AAPM TG‐235. Huang et al. also evaluated the use of 6 MV calibration for EBT3 film in Ir‐192 HDR brachytherapy and reported clinically acceptable dosimetric accuracy. ^25^ Film dosimetry is affected by several sources of uncertainty, including film nonuniformity, scanner response, calibration curve fitting, scan timing, orientation, positioning, image registration, and treatment delivery. These effects were mitigated through a rigorous calibration protocol and standardized scanning procedures. The methodology used was consistent with the film handling and calibration protocols described in Thatcher during HDR surface applicator commissioning and TG‐235.^23^, ^33^ ^,^A dedicated uncertainty analysis and uncertainty budget were also included to quantify the principal contributions from phantom fabrication, source positioning, treatment delivery, film calibration, scanning, registration, and data analysis. The dose profile and gamma analysis results should therefore be interpreted in the context of the combined measurement uncertainty.

Several limitations should be acknowledged. First, PLA does not fully reproduce the radiological properties of human tissues. Future studies could evaluate alternative materials with improved water or tissue equivalence while retaining the modular design. ABS, for example, may provide a density closer to water, although its use requires compatible printing hardware, adequate ventilation, and appropriate institutional safety measures. Composite filaments may further improve soft‐tissue or bone equivalence and enable fabrication of inserts spanning a broader range of radiological properties. Second, the 25% infill insert contains a patterned distribution of PLA and air rather than a homogeneous low‐density material. It therefore should not be considered equivalent to lungs, adipose tissue, or other specific biological tissue solely on the basis of its average physical density. Future work could incorporate homogeneous low‐density materials or independently characterized tissue‐equivalent composites to provide more anatomically representative test conditions. Third, the current phantom was designed primarily to represent a simplified interstitial brachytherapy configuration using needles. It does not accommodate shielded cylinder applicators, or T&O systems and therefore does not assess dose perturbations arising from high‐Z applicator components. This limits the phantom's ability to represent the broader range of clinical scenario in which MBDCAs may provide advantages over TG‐43 formalism. Future designs could incorporate modular component for high‐Z inserts, enabling assessment of applicator attenuation, shielding, and scatter effects. Fourth, the present study evaluated only AcurosBV. Application of the phantom to other MBDCAs, such as the Elekta Advanced Collapsed‐cone Engine, would improve the generalizability of the design and facilitate cross‐platform benchmarking.^31^ The current configuration was also limited to EBT4 film. Customized inserts could be developed to accommodate TLDs, OSLDs, or other point detectors, providing independent verification of film measurements, expanding detector compatibility, and potentially supporting external dosimetry audits. TLDs or OSLDs were not readily available at our institution and therefore were not evaluated in this study. Finally, the modular design may support applications beyond the specific E2E testing evaluated here. For example, it could serve as a practical educational platform for demonstrating the dosimetric effects of heterogeneities and the limitations of different dose calculation algorithms.^32^

Overall, the proposed phantom provides a low‐cost and adaptable platform for E2E evaluation of AcurosBV under controlled heterogeneous conditions. Its primary contribution is to complement the standardized test cases provided by AAPM TG‐186 and TG‐372 with an experimental assessment of the local imaging, planning, and delivery workflow. Further development using alternative materials, additional detector inserts, and broader applicator configurations may extend its value for commissioning, quality assurance, education, and collaborative validation.

## 5 CONCLUSION

This study demonstrated the feasibility of designing, fabricating, and validating of a novel, low‐cost, modular 3D‐printed phantom for E2E testing of MBDCAs in HDR brachytherapy. The phantom incorporated controlled heterogeneous inserts and provided a practical platform for assessing the complete workflow from CT imaging and treatment planning to dose delivery.

AcurosBV calculations showed close agreement with EBT4 film measurements, with gamma passing rates reaching 95% using 2%/2 mm criteria and dose profile differences below 2% in most evaluated directions. The observed differences between AcurosBV and TG‐43 should be interpreted in the context of the non‐water‐equivalent PLA phantom and the intentionally introduced heterogeneities rather than as a general comparison of the two calculation approaches.

The phantom's reproducible, modular, and customizable design offers an accessible measurement platform that can complement the standardized commissioning test cases described in AAPM TG‐186 and TG‐372. It may be particularly useful in resource‐limited settings, although further development is needed to incorporate additional detector types, radiologically equivalent materials, a broader range of heterogeneities, and more diverse applicator configurations.

## AUTHOR CONTRIBUTIONS

**Sean Grace**: Data curation; Formal analysis; Investigation; Visualization; Writing—original draft; Writing—review & editing. **Lin Wang**: Resources; Methodology; Validation; Writing—review & editing. **Yijian Cao**: Conceptualization; Resources; Supervision; Validation; Writing—review & editing. **Lyu Huang**: Conceptualization; Methodology; Data curation; Validation; Project administration; Supervision; Writing—review & editing.

## CONFLICT OF INTEREST STATEMENT

The authors declare no conflicts of interest related to this work.

## ETHICS STATEMENT

This study involved only phantom‐based dosimetric measurements and did not involve human participants, identifiable patient data, human tissue, or animals. Therefore, institutional ethics approval and informed consent were not required.

## Data Availability

The data that support the findings of this study are available from the corresponding author upon reasonable request.

## References

[1] Chargari C , Deutsch E , Blanchard P , et al. Brachytherapy: an overview for clinicians. CA Cancer J Clin. 2019;69(5):386–401. doi:10.3322/caac.21578 31361333

[2] Lim YK , Kim D . Brachytherapy: a comprehensive review. Prog Med Phys. 2021;32(2):25–39. doi:10.14316/pmp.2021.32.2.25

[3] Radcliffe BA , Kim Y , Raffi J , et al. Retrospective assessment of HDR brachytherapy dose calculation methods in locally advanced cervical cancer patients: acurosBV vs. AAPM TG43 formalism. J Appl Clin Med Phys. 2025;26(1):e14549. doi:10.1002/acm2.14549 PMC11712453 39382834

[4] Kuske RR . Breast brachytherapy. Hematol Oncol Clin North Am. 1999;13(3):543–558. 10.1016/s0889-8588(05)70074-5 10432428

[5] Siebert FA , Wolf S , Kóvacs G . Head and neck 192 Ir HDR‐brachytherapy dosimetry using a grid‐based boltzmann solver. J Contemp Brachytherapy. 2013;4:232–235. doi:10.5114/jcb.2013.39444 PMC3899639 24474973

[6] Kang H . Comparison of acuros BV and TG‐43 dose calculation for HDR skin brachytherapy with flap applicator in heterogeneous media. Med Phys. 2019;46(6):E113–E114.

[7] Bi S , Chen Z , Sun X , Dai Z . Dosimetric comparison of AcurosBV with AAPM TG43 dose calculation formalism in cervical intraductal high‐dose‐rate brachytherapy using three different applicators. Precis Radiat Oncol. 2022;6(3):234–242. doi:10.1002/pro6.1170

[8] Enger SA , Vijande J , Rivard MJ . Model‐based dose calculation algorithms for brachytherapy dosimetry. Semin Radiat Oncol. 2020;30(1):77–86. doi:10.1016/j.semradonc.2019.08.006 31727303

[9] Zourari K , Pantelis E , Moutsatsos A , et al. Dosimetric accuracy of a deterministic radiation transport based brachytherapy treatment planning system. Part I: single sources and bounded homogeneous geometries. Med Phys. 2010;37(2):649–661. doi:10.1118/1.3290630 20229874

[10] Petrokokkinos L , Zourari K , Pantelis E , et al. Dosimetric accuracy of a deterministic radiation transport based brachytherapy treatment planning system. Part II: monte Carlo and experimental verification of a multiple source dwell position plan employing a shielded applicator. Med Phys. 2011;38(4):1981–1992. doi:10.1118/1.3567507 21626931

[11] Zourari K , Pantelis E , Moutsatsos A , et al. Dosimetric accuracy of a deterministic radiation transport based^192^ Ir brachytherapy treatment planning system. Part III. Comparison to Monte Carlo simulation in voxelized anatomical computational models. Med Phys. 2013;40(1):011712. doi:10.1118/1.4770275 23298082

[12] Boman EL , Satherley TWS , Schleich N , Paterson DB , Greig L , Louwe RJW . The validity of Acuros BV and TG‐43 for high‐dose‐rate brachytherapy superficial mold treatments. Brachytherapy. 2017;16(6):1280–1288. doi:10.1016/j.brachy.2017.08.010 28967561

[13] Hofbauer J , Kirisits C , Resch A , et al. Impact of heterogeneity‐corrected dose calculation using a grid‐based Boltzmann solver on breast and cervix cancer brachytherapy. J Contemp Brachytherapy. 2016;2:143–149. doi:10.5114/jcb.2016.59352 PMC4873554 27257419

[14] Beaulieu L , Carlsson Tedgren Å , Carrier J , et al. TGR‐186: report of the task group 186 on model‐based dose calculation methods in brachytherapy beyond the TG‐43 formalism: current status and recommendations for clinical implementation. Med Phys. 2012;39(10):6208–6236. doi:10.1118/1.4747264 23039658

[15] Beaulieu L , Ballester F , Granero D , et al. AAPM WGDCAB Report 372: a joint AAPM, ESTRO, ABG, and ABS report on commissioning of model‐based dose calculation algorithms in brachytherapy. Med Phys. 2023;50(8):e946–60. doi:10.1002/mp.16571 37427750

[16] Sloboda RS , Morrison H , Cawston‐Grant B , Menon GV . A brief look at model‐based dose calculation principles, practicalities, and promise. J Contemp Brachytherapy. 2017;1:79–88. doi:10.5114/jcb.2017.65849 PMC5346608 28344608

[17] Madamesila J , McGeachy P , Villarreal Barajas JE , Khan R . Characterizing 3D printing in the fabrication of variable density phantoms for quality assurance of radiotherapy. Phys Medica PM Int J Devoted Appl Phys Med Biol Off J Ital Assoc Biomed Phys AIFB. 2016;32(1):242–247. doi:10.1016/j.ejmp.2015.09.013 26508016

[18] Zwierzchowski G , Bielęda G , Skowronek J , Mazur M . Film based verification of calculation algorithms used for brachytherapy planning‐getting ready for upcoming challenges of MBDCA. J Contemp Brachytherapy. 2016;8(4):326–335. doi:10.5114/jcb.2016.61828 PMC5018527 27648087

[19] Öllers MC , Swinnen ACC , Verhaegen F . Acuros^®^ dose verification of ultrasmall lung lesions with EBT‐XD film in a homogeneous and heterogeneous anthropomorphic phantom setup. Med Phys. 2020;47(11):5829–5837. doi:10.1002/mp.14485 32970849

[20] Pappas EP , Zoros E , Moutsatsos A , et al. On the experimental validation of model‐based dose calculation algorithms for 192Ir HDR brachytherapy treatment planning. Phys Med Biol. 2017;62(10):4160. doi:10.1088/1361-6560/aa6a01 28353447

[21] Antunes PCG , Siqueira PdeTD , Shorto JMB , Yoriyaz H . Heterogeneous physical phantom for I‐125 dose measurements and dose‐to‐medium determination. Brachytherapy. 2024;23(1):73–84. doi:10.1016/j.brachy.2023.08.007 38016863

[22] Low DA , Harms WB , Mutic S , Purdy JA . A technique for the quantitative evaluation of dose distributions. Med Phys. 1998;25(5):656–661. doi:10.1118/1.598248 9608475

[23] Niroomand‐Rad A , Chiu‐Tsao S , Grams MP , et al. Report of AAPM task group 235 radiochromic film dosimetry: an update to TG‐55. Med Phys. 2020;47(12):5986–6025. doi:10.1002/mp.14497 32990328

[24] Iaea T , 1585 Measurement uncertainty: A practical guide for secondary standards dosimetry laboratories. Published online 2008.

[25] Huang L , Gaballa H , Chang J . Evaluating dosimetric accuracy of the 6 MV calibration on EBT3 film in the use of Ir‐192 high dose rate brachytherapy. J Appl Clin Med Phys. 2022;23(5):e13571. doi:10.1002/acm2.13571 PMC9121041 35226398

[26] Sorriaux J , Kacperek A , Rossomme S , et al. Evaluation of gafchromic® EBT3 films characteristics in therapy photon, electron and proton beams. Phys Med. 2013;29(6):599–606. doi:10.1016/j.ejmp.2012.10.001 23107430

[27] Tien CJ , Chen ZJ . Deployment and performance of model‐based dose calculation algorithm in 192Ir shielded cylinder brachytherapy. Brachytherapy. 2019;18(6):883–889. doi:10.1016/j.brachy.2019.07.006 31444132

[28] DeWerd LA , Ibbott GS , Meigooni AS , et al. TG‐138: a dosimetric uncertainty analysis for photon‐emitting brachytherapy sources: report of AAPM task group no. 138 and GEC‐ESTRO. Med Phys. 2011;38(2):782–801. doi:10.1118/1.3533720 PMC3033879 21452716

[29] Palmer A , Hayman O , Ioannou L , Nagar YS . PO‐332 Is HDR equipment performance suitable for modern brachytherapy? Positional errors, dosimetric impact & case study. Radiother Oncol. 2012;103:S132–S133.

[30] ISO. I, OIML B . Guide to the Expression of Uncertainty in Measurement . 1993. Accessed September 21, 2026. http://chapon.arnaud.free.fr/documents/resources/stat/GUM.pdf

[31] Howie A , Poder J , Brown R , et al. Comparison of TG43 and hounsfield unit‐based TG186 brachytherapy dose metrics in oncentra brachy for 100 patients receiving interstitial partial breast irradiation. Brachytherapy. 2021;20(3):655–663. 10.1016/j.brachy.2020.11.013 33358142

[32] Tino R , Yeo A , Leary M , Brandt M , Kron T . A systematic review on 3D‐printed imaging and dosimetry phantoms in radiation therapy. Technol Cancer Res Treat. 2019;18:1533033819870208. 10.1177/1533033819870208 PMC6856980 31514632

[33] Thatcher TR , HDR Brachytherapy for Skin Cancer Using H.A.M. Applicator. University of Nevada, doi:10.34917/19412182

